# Novel in ovo application of green-synthesized Fe_3_O_4_, Cu, and ZnO nanoparticles: enhancing early growth and physiological responses in Domiaty ducks

**DOI:** 10.1038/s41598-026-62297-6

**Published:** 2026-07-22

**Authors:** Khaled Sayed-Ahmed, Nabil A. Azzaz, Sahar E. Hamed, Heba H. Nekshara, Eman A. El-Said

**Affiliations:** 1 Department of Agricultural Biotechnology, The Center for Excellence in Research of Advanced Agricultural Sciences, Faculty of Agriculture, Damietta University, New Damietta, 34517 Egypt; 2 Department of Chemistry, Faculty of Science, New Mansoura University, New Mansoura, 35522 Egypt; 3 Department of Animal, Poultry and Fish Production, Faculty of Agriculture, Damietta University, New Damietta, 34517 Egypt

**Keywords:** Domiaty ducks, Copper, Iron oxide, Zinc oxide, Nanoparticles, In ovo, Embryonic injection, Biochemistry, Biotechnology, Nanoscience and technology, Physiology, Zoology

## Abstract

Enhancing embryonic nutrient utilization is a key strategy for improving the early growth and health of poultry. This study investigated the effects of in ovo injection (30 µg/egg) of green-synthesized iron oxide nanoparticles (Fe_3_O_4_-NPs), copper nanoparticles (Cu-NPs), and zinc oxide nanoparticles (ZnO-NPs) on the early growth performance, physiological responses, antioxidant status, and intestinal morphology of Domiaty ducklings. The nanoparticles (NPs) were characterized by transmission electron microscopy (TEM), X-ray diffraction (XRD), and zeta potential analyses, confirming their spherical morphology, nanoscale size (2–16 nm), and good stability. A total of 450 fertile duck eggs were assigned to five groups: non-injected control, saline-injected control, and three NPs-treated groups. In ovo administration was performed on the 14th embryonic day (ED). The results showed significant improvements in hatchling body weight and 7-day post-hatch growth in all NPs-treated groups, with Fe_3_O_4_-NPs and ZnO-NPs producing the greatest responses. Serum lipid profiles showed favorable changes, with reduced cholesterol, triglyceride (TG), and low-density lipoprotein (LDL) levels, and increased high-density lipoprotein (HDL) levels. Hemoglobin (Hb), transferrin (Tf), thyroid hormones (T3 and T4), IgG levels, and antioxidant indicators, such as total antioxidant capacity (TAC), superoxide dismutase (SOD), and glutathione (GSH), were enhanced, whereas malondialdehyde (MDA) levels were reduced. Histological examination revealed improved jejunal villus development and crypt architecture. In general, the findings reveal that in ovo administration of biosynthesized metal NPs may positively influence early growth, selected physiological and immune-related traits, antioxidant status, and intestinal development in Domiaty ducks.

## Introduction

The Domiaty duck is an indigenous Egyptian breed of economic importance, with potential for genetic improvement. As a native genetic resource, the Domiaty duck is well adapted to local environmental conditions and contributes to a sustainable poultry production system while representing a valuable reservoir of genetic diversity^1,2^. However, compared with highly selected commercial breeds, indigenous duck populations generally exhibit lower growth performance, emphasizing the need for effective nutritional strategies to enhance their productivity without compromising their adaptive characteristics. Previous studies have focused on the distinctive productive and genetic traits of the Domiaty duck, supporting its value for breeding and improvement programs^3^. Early nutritional programming through in ovo intervention has emerged as a promising strategy for enhancing embryonic development, intestinal maturation, and post-hatch performance in poultry^4,5^. However, previous studies on in ovo nutrition and nano-mineral supplementation have focused on commercial poultry genotypes, whereas information on indigenous duck breeds remains scarce.

Duck embryos exhibit developmental patterns that differ from those of chickens, particularly in terms of incubation length, embryonic nutrient utilization, and maturation of the gastrointestinal tract and associated physiological systems. Therefore, nutritional interventions during embryogenesis may have species-specific effects on post-hatch growth, intestinal development, and physiological performance, emphasizing the importance of investigating these strategies in duck populations^6^.

Improving embryonic metabolism in avians has become a central focus in modern poultry production, as embryonic development relies heavily on the nutrient reserves deposited in the egg by the breeder hens. The yolk sac serves as the primary nutrient source throughout incubation, and its efficient utilization determines hatchability, chick quality, and early post-hatch performance^7^. Therefore, the metabolic rate of the embryo is closely linked to both the quantity and bioavailability of nutrients stored in the egg, as well as to the embryo’s capacity to mobilize and absorb these nutrients during critical stages of development^7,8^. To enhance embryonic nutrition beyond what is naturally deposited in the egg, in ovo injection technology has been developed as a strategic tool for supplying exogenous nutrients directly to the developing embryo^9,10^.

In ovo injection was performed on embryonic day 14, as this represents a critical stage of duck embryonic development. Given the approximately 28-day incubation period of ducks, major organogenesis is largely completed by this stage, while the chorioallantoic membrane is highly vascularized and the gastrointestinal, hepatic, and immune systems undergo rapid functional maturation. These developmental features facilitate the efficient absorption and utilization of the injected nutrients and bioactive compounds. Moreover, the mid-incubation period is characterized by increased metabolic demands and rapid tissue growth, making nutritional interventions particularly effective. Earlier injection may interfere with embryonic development and increase mortality, whereas later administration may limit the time available for physiological responses before hatching. Therefore, embryonic day 14 provides an appropriate window for in ovo nutritional intervention to support embryonic development and post-hatch performance^4,5,11,12^.

In recent years, attention has shifted toward nanotechnology in poultry nutrition. NPs, typically ranging from 1 to 100 nm in size, possess unique physicochemical properties, including increased surface area, enhanced reactivity, and improved solubility, compared to their conventional counterparts. These characteristics may enhance mineral bioavailability and biological activity at lower inclusion levels^13–15^. The application of nano-minerals in broiler diets has shown promising effects on growth performance, antioxidant status, immune responses, and carcass quality^16^. When delivered via in ovo injection, nanosized nutrients may further support early gut development and immunophysiological maturation^17^.

Among trace minerals, iron, zinc, and copper are essential for normal embryonic growth and physiological development of the embryo. Iron is a critical component of hemoglobin and numerous enzymes involved in oxygen transport and cellular respiration^18,19^. Iron at the nanoscale may enhance its uptake and cellular utilization owing to the increase in its surface area and reactivity due to its small size^20,21^. Additionally, it may minimize heme breakdown in the spleen and increase the lifespan of red blood cells, leading to higher hemoglobin levels^22^.

Copper contributes to angiogenesis, hemoglobin synthesis, and redox balance and acts as a cofactor for enzymes such as superoxide dismutase^23^. Adequate copper supply has also been linked to improved lipid metabolism and enhanced antioxidant capacity in poultry^24^. Zinc is of particular interest in embryonic development because of its involvement in numerous metalloenzymes and transcription factors. It functions as a structural and catalytic cofactor that influences cellular differentiation and tissue morphogenesis^25^. Maternal dietary zinc supplementation or in ovo zinc administration has been shown to increase zinc deposition in the yolk and facilitate its transfer to the embryo, thereby supporting growth and enhancing antioxidant defense mechanisms^26,27^.

Plant-mediated synthesis has attracted considerable interest because of its simplicity and safety profile. For example, extracts from *Stevia rebaudiana* contain steviol glycosides and other phytochemicals that can reduce metal ions and stabilize NPs^28^. The biological activity of these NPs depends largely on their size, surface characteristics, and chemical composition, which influence their reactivity and interactions with biological tissues^29^. Despite promising results, further research is required to optimize NPs synthesis methods, determine safe and effective dosages, and establish the most appropriate timing for in ovo administration. The dynamic process of mineral mobilization from the yolk sac to embryonic tissues suggests that timing is critical for maximizing bioavailability and functional impact^30^. Moreover, understanding the molecular mechanisms underlying nano-mineral action, particularly their influence on antioxidant systems, gene expression, and immune modulation, remains an important area of investigation.

Trace minerals play essential roles in duck growth, hematopoiesis, antioxidant defense, and immune functions. Recent studies have demonstrated that optimizing trace mineral nutrition can improve the productive performance and physiological status of ducks, and the enhanced bioavailability of nano-trace minerals may further increase their biological efficiency^31^.

Despite the increasing interest in in-ovo nutritional interventions, most previous studies have been conducted in commercial broiler chickens, whereas information regarding indigenous duck breeds is limited. Native duck populations may differ in embryonic development, nutrient metabolism, and physiological responses, highlighting the need for species-specific studies. Therefore, evaluating the effects of biosynthesized nano-trace minerals in Domiaty ducks may contribute to the development of sustainable nutritional strategies for native duck production. However, compared with highly selected commercial duck breeds, such as Cherry Valley, indigenous duck populations generally exhibit lower growth performance and have received considerably less attention regarding advanced nutritional interventions. Therefore, evaluating the effects of green-synthesized Fe_3_O_4_-NPs, Cu-NPs, and ZnO-NPs in Domiaty ducks addresses an important acknowledged gap and may provide a sustainable nutritional approach for improving intestinal development and growth performance, while supporting the productive efficiency of this valuable native Egyptian genetic resource.

This study aimed to enhance embryonic nutrient availability through in ovo injection of Fe_3_O_4_-NPs, Cu-NPs, and ZnO-NPs as a promising strategy for improving broiler productivity and health. In this respect, the effect of in ovo injection of the prepared NPs on the growth parameters of Domiaty ducks was evaluated. In addition, several blood biochemical indicators and serum and hepatic antioxidant activities were determined. Furthermore, microscopic histological examination of the jejunum was performed to study the effects of the prepared NPs type and size on the physiological and morphological alterations in the injected groups. This study revealed an outstanding approach to enhance the productivity, immunity, and overall health of the Domiaty duck strain, which is the most important domestic duck strain in Egypt.

## Experimental sections

### Materials

Fresh leaves of *Stevia rebaudiana* were obtained from a commercial source in Zagazig City, Al-Sharqia Governorate, Egypt. Whatman No. 1 filter paper, potassium ferricyanide, trichloroacetic acid, ferric chloride, ascorbic acid, ferrous chloride, sodium hydroxide, copper nitrate, and zinc nitrate were purchased from Sigma-Aldrich.

### Procedures

#### Preparation of plant extract

Fresh leaves of *S. rebaudiana* were washed using sterilized distilled water and then left in the laboratory shade at room temperature (25 ± 2 ˚C) for approximately one week until dry. Dried leaves (5 g) were added to 200 mL of distilled water and boiled for 10 min. The resulting solution was filtered using cotton, followed by Whatman No.1 filter paper to discard the plant residues. The samples were then placed in a closed flask and stored at 4 °C for further use.

### Green synthesis of NPs

#### Preparation of Fe_3_O_4_-NPs

Ferrous chloride (2 mM) and ferric chloride (1 mM) at a molar ratio of 2:1 were used as precursors for Fe_3_O_4_-NPs. In addition, an aqueous extract of *S. rebaudiana* (100 mL) was used as an eco-friendly reducing and capping agent and added to the previous mixture (120 mL) at a volume ratio of 1:1.2, under continuous stirring for 10 min at room temperature (25 ± 2 °C). Subsequently, 1 N NaOH was added to adjust the pH to 11 under continuous stirring for 45 min. The solution then turned black, indicating the successful formation of Fe_3_O_4_-NPs. The resulting solution was centrifuged at 10,000 rpm for 10 min. The supernatant was discarded, and the precipitate was collected with a yield of approximately 27.8 mg. Finally, the prepared Fe_3_O_4_-NPs were dried at 60 °C for 6 h and stored in a closed container until further characterization^32^.

#### Synthesis of Cu-NPs

Cu-NPs were prepared via a green method using copper nitrate as a precursor and *S. rebaudiana* extract as a reductant and stabilizer. The plant extract was mixed with 10 mM copper nitrate at a volume ratio of 1:10 under continuous stirring, followed by dropwise addition of 1 N sodium hydroxide to adjust the pH to 9. The mixture was then boiled for 24 h under reflux conditions until a reddish-brown color was observed, indicating the formation of Cu-NPs. The obtained solution was centrifuged at 10,000 rpm for 10 min, and the precipitate was dried in Petri dishes at 45 °C overnight and ground for further use^33^.

#### Preparation of ZnO-NPs

ZnO-NPs were synthesized via a precipitation reaction between zinc nitrate and *S. rebaudiana* extract. Zinc nitrate (2.5 g) was dissolved in 100 mL of an aqueous *S. rebaudiana* extract. The mixture was then ultrasonicated for 30 min at room temperature (25 ± 2 °C). The solution was centrifuged at 300 rpm for 5 min. The white precipitate was calcined at 400 °C for 3 h and then stored at 4 °C for further use^34^.

### NPs characterization

#### Transmission electron microscopy (TEM) analysis

The NPs were examined using a transmission electron microscope (200 kV; JEOL JEM 2100 F, Tokyo, Japan) to characterize their morphology and diameters. Specimens were prepared for TEM analysis using a drop obtained from the colloidal solutions of the NPs. A 400-mesh copper grid was used to load the drop and was coated with a carbon film as an amorphous layer. The solvent was then evaporated at room temperature (25 ± 2 ˚C)^35^. Approximately 150 particles were used to determine the size range of the prepared NPs and for the histogram of the synthesized NPs sizes based on the obtained scale bar in each micrograph.

#### X-ray diffraction (XRD) and zeta potential analyses

XRD analysis was performed to study the crystalline nature of the NPs using an X-ray diffractometer (Bruker D8 ADVANCE, Karlsruhe, Germany) to confirm the formation of the Fe_3_O_4_-NPs, Cu-NPs, and ZnO-NPs. The tube target of the X-rays was Cu, with a current and voltage of 30 mA and 40 kV, respectively. In addition, zeta potential values of the colloidal solutions of the prepared NPs were measured using a zetasizer (Zetasizer Nano ZS90, Malvern, UK) to evaluate the stability of the colloidal solutions of the prepared NPs. Then, the size of the prepared NPs was also determined using.

The average crystallite size of the synthesized NPs was also determined from the XRD pattern using the Debye–Scherrer equation, which relates the broadening of the diffraction peaks to the crystallite size. The crystallite size was calculated using the following equation:

$$\:D=\frac{K\lambda\:}{\beta\:\:\mathrm{cos}\theta\:}$$

where D is the average crystallite size (nm), K is the shape factor (~ 0.9), λ is the X-ray wavelength (1.5406), β is the full width at half maximum (FWHM) of the diffraction peak expressed in radians, and θ is the Bragg diffraction angle. The crystallite size was calculated from the most intense diffraction peak, and the average value was reported as the crystallite size of the prepared NPs^36^.

#### Experimental design (in ovo injection and incubation)

The current study was conducted at the Agricultural Biotechnology Department and Animal, Poultry, and Fish Department, Faculty of Agriculture, Damietta University, Egypt. A total of 450 fertile duck eggs (67.0 ± 0.58 g) were obtained from the El-Serw Poultry Research Station, Animal Production Research Institute, Agricultural Research Center, Ministry of Agriculture, Egypt. The experimental protocol was approved by the Research Ethics Committee of Damietta University (Subcommittee for the Basic Sciences Sector, Faculties of Science and Agriculture), Egypt (DuRec No. 296; approval date: December 2, 2025). All animal experiments were conducted in accordance with the Guide to Academic Integrity and Research Ethics issued by the Supreme Council of Universities (SCU), Egypt, the institutional guidelines and regulations for the care and use of experimental animals, and the relevant national ethical standards.

Duck eggs were randomly divided into five equal groups. The first group was kept as a negative control (without injection), the second (positive control) was injected with 0.1 mL saline solution 0.9%, while the third, fourth, and fifth groups were injected with Fe_3_O_4_-NPs, Cu-NPs, and ZnO-NPs, respectively, at a concentration of 30 µg/egg^37,38^.

*In ovo* injections were performed on the 14th embryonic day (ED) into the yolk sac at the broad end of the egg using a 24G hypodermic needle (25 mm long), following the technique described by^39^. Embryonic day 14 was selected as the injection time point to ensure the efficient uptake of the injected substances while minimizing the risk of embryonic injury. The entire injection procedure was performed under a laminar flow system (Model: Bio air SAFEMATE EZ model PRIME 1.2). Following injection, the puncture site was sealed with sterile paraffin wax, and the eggs were immediately returned to the incubator. The incubation conditions were maintained at a temperature of 99–100 °F and a relative humidity of 55–60% throughout the first 24 days of incubation. The eggs were automatically turned at 1-h intervals until day 24, after which turning was discontinued, and the eggs were transferred to the hatcher. During the hatching period, the temperature was adjusted to 98–99 °F, and the relative humidity was increased to 70% until hatching^12^.

#### Ducklings brooding, rearing, and management

After hatching, all ducklings from each treatment group were individually weighed and randomly allocated to three replicates. All ducklings were reared under identical management and hygienical conditions. The brooding temperature was maintained at 32–33 °C during the first two days’ post-hatch and gradually reduced by 0.5 °C per day until reaching 30 °C. Ducklings were housed at a stocking density of 4.5 birds/m², and gas heaters were used to maintain the required temperature. Continuous lighting was provided throughout the experimental period, and feed and water were offered ad libitum. At 7 days of age, ten ducklings were randomly selected from each experimental group, individually weighed, and slaughtered for sample collection. The livers were carefully weighed, and the relative liver weights were calculated and expressed as percentages of the live body weights of each duckling.

#### Blood biochemical indicators

From this duckling six blood samples from each group at 7 days’ post-hatch were randomly collected after slaughter, and hemoglobin (Hb) was determined using Auto hematology analyzer (Rayto, model: RT7600S; Guangdong, China), while transferrin (TF) levels was determined using commercial kit (Spectrum Diagnostic Kits, Spec. Corp. Egypt), then blood samples were collected from the jugular vein of six ducks from each group, placed in coagulant tubes, and centrifuged at 3500 rpm for 15 min at room temperature to obtain serum. After separation, the serum was stored at -20 °C until analysis. Serum concentrations of total cholesterol were determined using the enzymatic colorimetric method^40^, and triglycerides^41^, high-density lipoprotein (HDL)^42^, and low-density lipoprotein (LDL) were determined^43^. Moreover, liver enzyme activities, including alanine aminotransferase (ALT), aspartate transaminase (AST), and alkaline phosphatase (ALP), were determined using the methods described by^44,45^, and immunoglobulins G, including immunoglobulin A (IgA), immunoglobulin M (IgM), and immunoglobulin G (IgG) values were determined using ELISA kits according to^46^. Thyroid Hormones, including triiodothyronine (T3) and thyroxine (T4), were quantified using commercial diagnostic kits for serum analysis according to^47,48^. In addition, antioxidant status, such as TAC^49^, SOD^50^, MDA^51^, and GSH^52^_,_ was determined.

#### Tissue collection for TAC in liver examination

Liver samples (50 mg of liver at 7days old) were collected from six ducks in each treatment group after slaughter. Liver tissue was homogenized to a 10% liver homogenate solution in precooled 0.85% normal saline and centrifuged at 3,500 rpm for 15 min at 4 °C (Centrifuge 5804R; Eppendorf, Hamburg, Germany) to remove the cell debris and nuclei. The supernatants were collected and stored at − 80 °C for TAC content analysis, according to the method described by^53^.

#### Histological observation

Immediately after slaughter (7 days old), samples (three specimens, each approximately 1 cm from the locations) were collected and prepared after staining with hematoxylin and eosin using standard paraffin embedding procedures. The jejunum was evaluated in each section in all groups using the image processing and analysis system of the software, Axio Vision, specialized for the microscope according to^54^.

#### Statistical analysis

Statistical analyses were conducted to evaluate the effects of injecting Domiaty duck embryos with different types of NPs. Data were analyzed using SAS (2006). One-way analysis of variance (ANOVA) was performed to assess the differences between experimental groups. For post hoc comparisons, Duncan’s multiple range test was applied, as it is widely used in biological and agricultural experiments to identify significant differences between treatment means while maintaining sensitivity to variability of groups. Statistical significance was set at *p* < 0.05^55^. The following model was used:

$${{\mathrm{K}}_{{\mathrm{ij}}}} = \mu {\text{ }} + {\text{ }}{{\mathrm{G}}_{\mathrm{i}}} + {\pounds_{{\mathrm{ij}}}}$$

where, K_ij_ is the dependent variable of the j Domiaty ducklings in the treatments, µ is the overall mean, G_i_ denotes the effect of the treatments (i: 1, 2, 3, 4, and 5), and £_ij_ is the experimental random error component.

The sample size for each analysis was selected according to the requirements of the respective measurements and is specified throughout this study.

## Results and discussion

### Characterization of the prepared NPs

#### TEM analysis

**Fig. 1:**
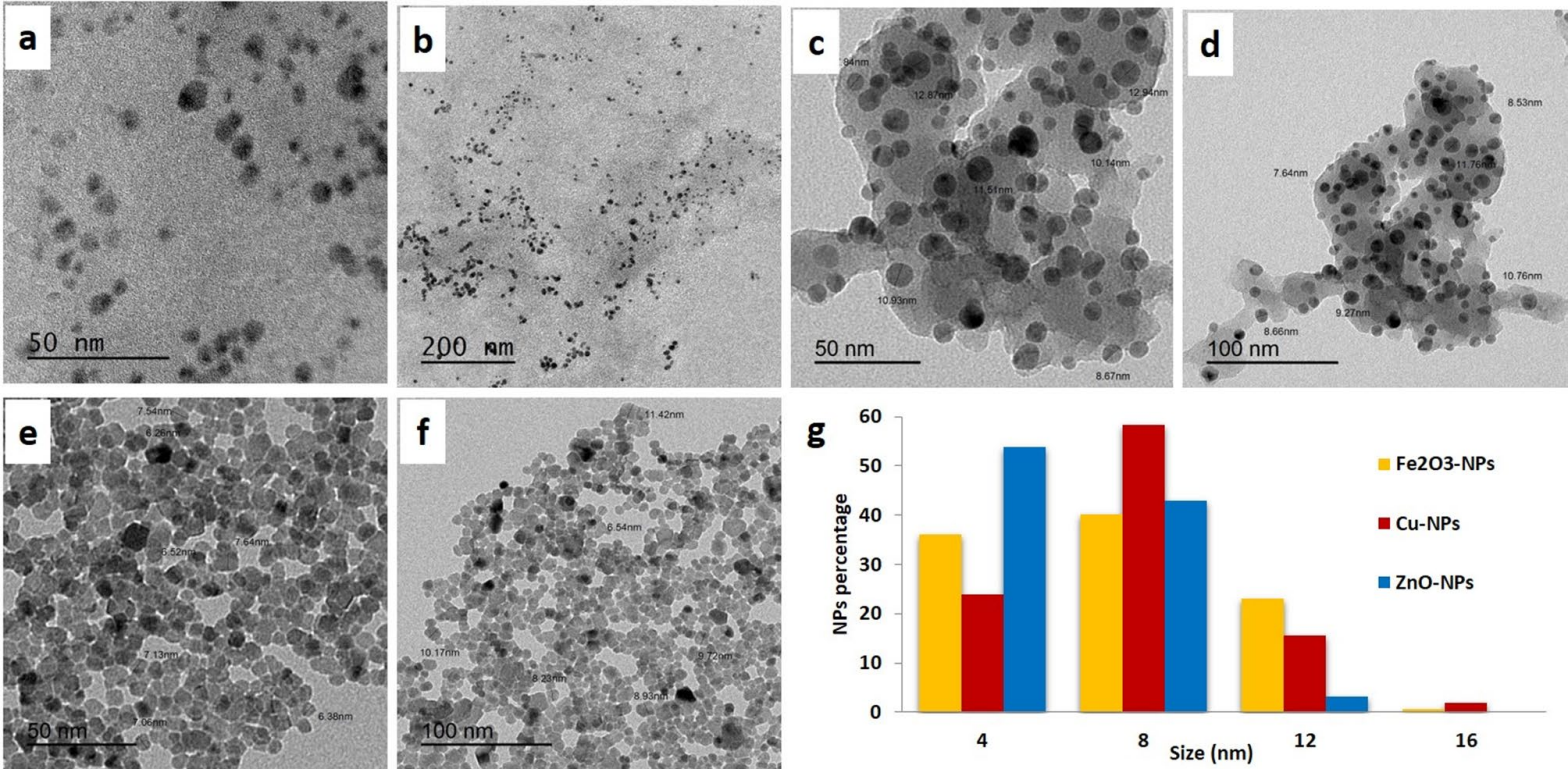
TEM micrographs of (**a**,**b**) Fe_3_O_4_-NPs, (**c**,**d**) Cu-NPs, (**e**,**f**) ZnO-NPs, (**g**) histogram of the prepared NPs.

TEM analysis was conducted to confirm the formation of Fe_3_O_4_-NPs, Cu-NPs, and ZnO-NPs at the nanoscale (Fig. 1). The prepared NPs were in the range of 2–16, 3–14, and 4–16 nm, respectively. The TEM micrographs of the synthesized NPs revealed their spherical and monodisperse nature. All of them were well distributed in the colloidal solution that was loaded onto the grid. The majority of ZnO-NPs and Cu-NPs were approximately 4 and 8 nm in size, respectively. In addition, Fe_3_O_4_-NPs were the most monodisperse NPs compared to the other prepared NPs, as shown in the histogram of the prepared NPs (Fig. 1(g). The histogram included bins 4 nm wide, centered at 4, 8, 12, and 16 nm. NPs with sizes of 6–10 nm were considered collectively as NPs with an average size of 8 nm. The obtained TEM micrographs confirmed the successful synthesis of Fe_3_O_4_-NPs, Cu-NPs, and ZnO-NPs with the desired morphology and size.

The TEM micrographs of the Fe₃O₄-NPs (Fig. 1a, b) show that the NPs appeared surrounded by a faint low-contrast boundary and showed limited direct particle fusion, which may indicate the presence of an organic layer adsorbed onto the NPs surfaces. This behavior reveals that phytochemical constituents originating from *S. rebaudiana* extract acted as capping and stabilizing agents during NPs synthesis, reducing excessive particle aggregation and promoting NPs stabilization. Similarly, the TEM images of Cu-NPs (Fig. 1c, d) demonstrated nearly spherical particles with a visible difference in electron density between the darker NPs cores and the surrounding lighter region. These features may be attributed to the adsorption of biomolecules from the plant extract onto the Cu-NPs surface, forming a thin organic capping layer that restricts particle growth and enhances colloidal stability^56^.

#### XRD analysis

**Fig. 2:**
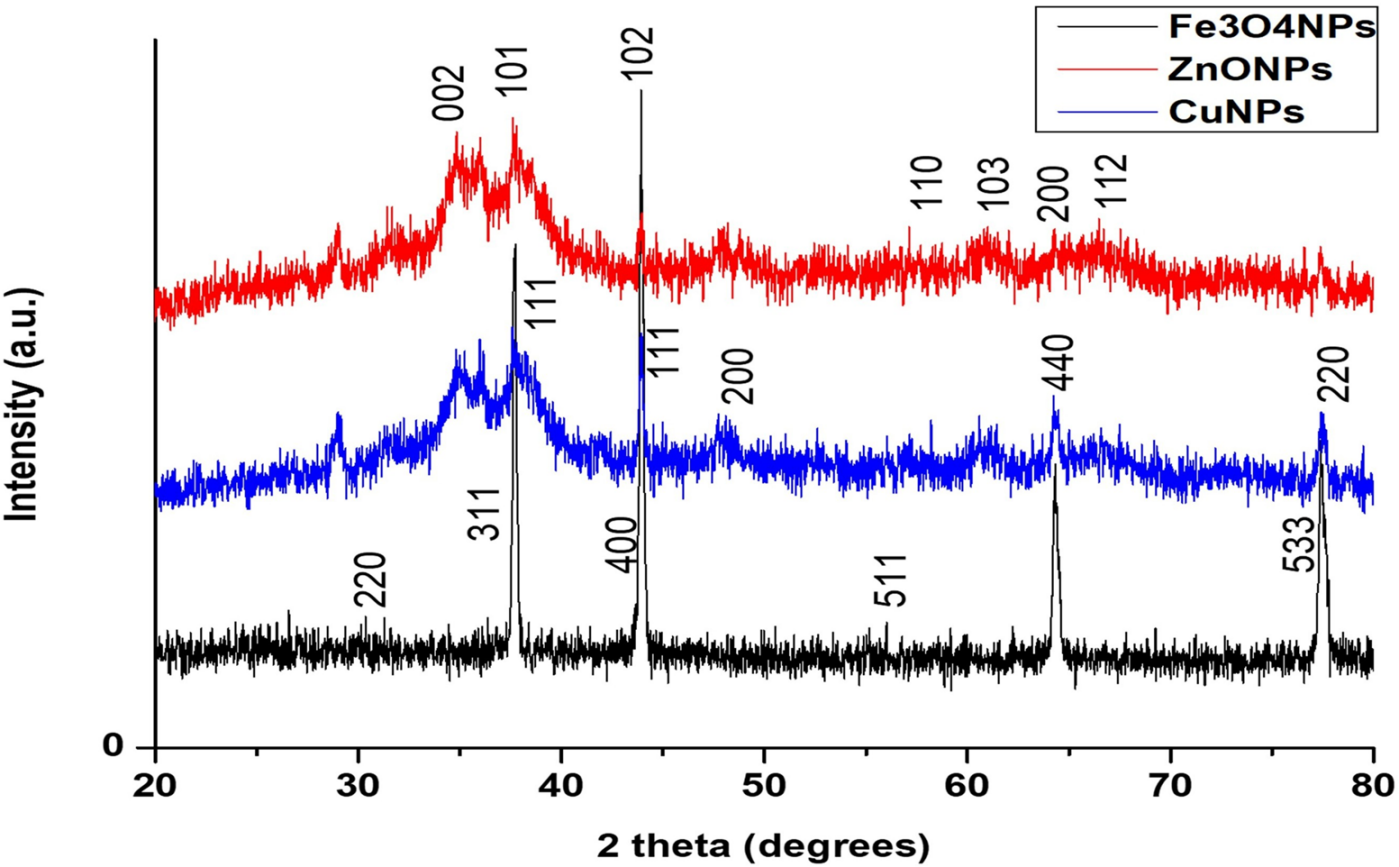
XRD patterns of Fe_3_O_4_-NPs, Cu-NPs, and ZnO-NPs.

XRD patterns of Fe_3_O_4_-NPs, Cu-NPs, and ZnO-NPs were obtained to ensure the formation of these NPs, as shown in Fig. 2. All of them were highly crystalline and exhibited several peaks, indicating the corresponding crystal planes. The diffraction peaks of the Fe_3_O_4_-NPs were (220), (311), (400), (511), (440), and (533) planes at 2θ values of 30.1°, 37.74°, 43.94°, 57.0°, 64.32°, and 77.4 °, respectively^57–59^. Among these reflections, the peak assigned to the (311) plane exhibited the highest intensity, indicating that this plane represents the predominant crystallographic orientation of the synthesized NPs. The presence of well-defined diffraction peaks without detectable secondary phases suggests high phase purity and the successful formation of magnetite NPs.

Additionally, powder X-ray diffraction was used to analyze the phase crystallinity and structural composition of the produced Cu-NPs. The diffraction pattern of the Cu-NPs showed peaks at 37.64°, 43.94°, 50.3°, 64.48°, and 77.56° 2-theta values corresponding to the (111), (111), (200), (220), and (220) crystal planes, respectively. Each diffraction peak aligned well with the typical pattern for Cu-NPs (JCP DS No. 040836) and matched the previous literature^60,61^. The very strong peak intensities indicate a high degree of crystallinity in the sample, and the broadening of the peak is a result of the nanocrystalline structure^61^.

The diffraction pattern of ZnO-NPs in which the peaks at 2θ values of 34.84°, 36°, 47.48°, 56.12°, 64.22 °, 66.7° and 67.54° correspond to (002), (101), (102), (110), (103), (200) and (112) planes, respectively, which is similar to JCPDS Card No. 01-079-2205 and agrees with the available literature of^62–64^.

The average crystallite size of the synthesized NPs was also determined from the XRD pattern using the Debye–Scherrer equation. The crystallite sizes of the prepared Fe_3_O_4_-NPs, Cu-NPs, and ZnO-NPs were approximately 6.32, 4.6, and 6.3 nm, respectively, which is consistent with the results obtained from the TEM micrographs shown in Fig. 1. Although the sizes obtained by XRD and TEM were not exactly identical, this behavior is expected because the two techniques characterize different aspects of the prepared NPs. TEM determines the physical particle size, including the entire visible NPs dimensions and any surrounding amorphous or surface-associated layers, whereas XRD estimates the average crystallite size, corresponding only to the coherent crystalline domains responsible for the diffraction. In addition, the surface stabilization effects of phytochemicals or capping molecules remaining after synthesis may cause this variation. Therefore, a single nanoparticle observed by TEM may consist of one or several crystallites, leading to slight differences between the two measurements^65^. In general, the XRD-derived crystallite sizes were slightly smaller than or close to the dominant TEM particle sizes, indicating that the prepared NPs were predominantly composed of single-crystalline or nearly single-crystalline domains with limited aggregation. The close agreement between the TEM and XRD results confirmed the successful synthesis of well-dispersed nanocrystalline Fe_3_O_4_-NPs, Cu-NPs, and ZnO-NPs with dimensions on the nanometer scale.

#### Zeta potential

**Fig. 3:**
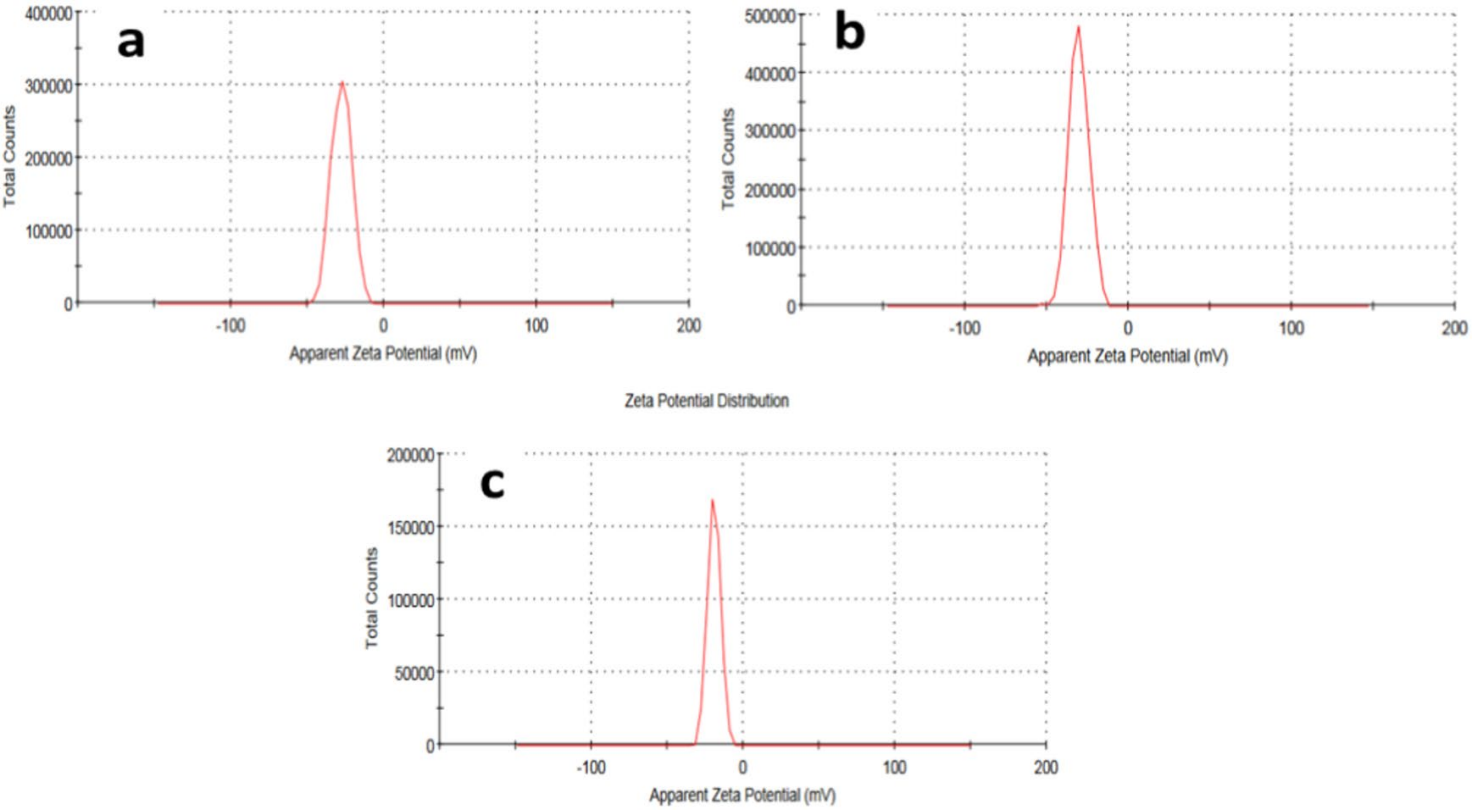
Zeta potentials of Fe_3_O_4_-NPs, Cu-NPs, and ZnO-NPs synthesized using SE.

Zeta potential analysis was conducted to examine the stability of the colloidal solutions of the synthesized Fe_3_O_4_-NPs, Cu-NPs, and ZnO-NPs. The results demonstrated that the NPs surfaces had a negative charge. The calculated zeta potentials of the NPs were − 27.7, -30.4, and − 19.2 mV, respectively. (Fig. 3). The obtained results illustrated that the biomolecular coating on the prepared NPs enhanced their stability by opposing forces between the NPs, and therefore, prevented their aggregation^66^.

### Biological behavior of the prepared NPs

The observed variations in hatchability percentage among experimental groups may be partially attributed to the physiological effects of in ovo administration of nano-trace minerals. In ovo injection at the embryonic developmental stage provides a direct route for nutrient delivery, bypassing maternal limitations in nutrient deposition into the egg, and ensuring immediate availability during critical phases of embryogenesis. In the present study, hatchability was 85.6% in the control group and slightly reduced in the positive control group (80%). However, the nano-mineral treatments improved or maintained hatchability, reaching 86.7%, 84.4%, and 83.3% in the Fe_3_O_4_-NPs, Cu-NPs, and ZnO-NPs groups, respectively. Additionally, early embryonic mortality ranged from 5.56% to 10.00%, with the lowest value observed in the positive control group (5.56%) and the highest in the ZnO-NPs group (10.00%). Late embryonic mortality was highest in the positive control group (14.44%), whereas the Fe_3_O_4_-NPs treatment exhibited the lowest rate (5.56%). Both the Cu-NPs and ZnO-NPs groups showed an identical late embryonic mortality rate of 6.67%, compared to 7.78% in the negative control group. This improvement may be associated with enhanced bioavailability and cellular uptake of nanosized minerals, thereby facilitating their more efficient utilization during hematopoiesis, organogenesis, and metabolic regulation. Nano-form trace minerals have been reported to enhance antioxidant defense systems and reduce embryonic oxidative stress, a key factor influencing embryo mortality during incubation. Therefore, in ovo supplementation may improve embryonic survival by stabilizing redox balance and supporting mitochondrial function during late embryonic development. These findings are consistent with previous reports indicating that in ovo injection of bioavailable nutrients improves embryonic viability and hatchability by optimizing nutrient availability during critical developmental windows^67^.

#### Growth performance

**Fig. 4:**
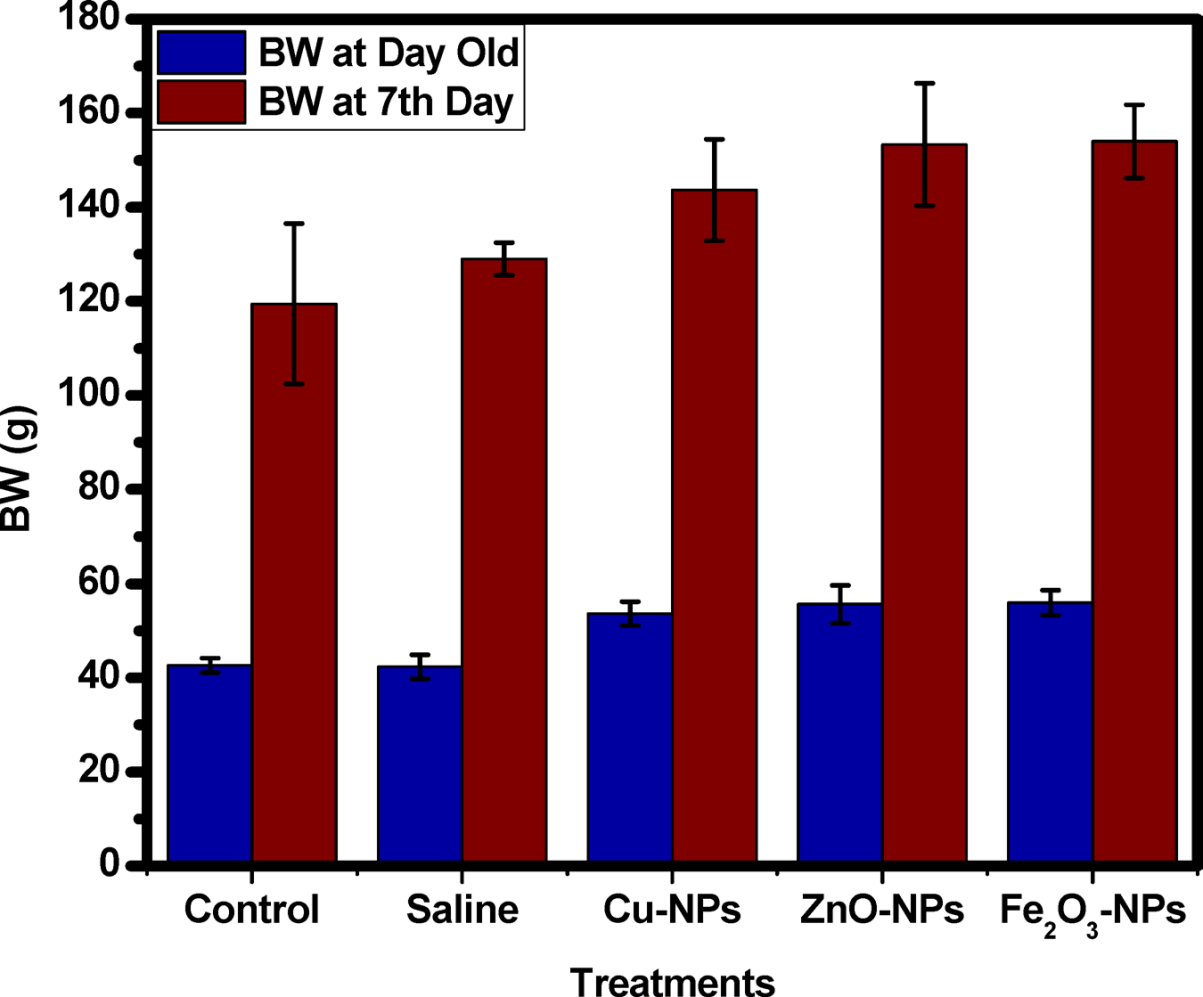
The BW on the day-old hatchlings and the 7th day of duck chicks hatched from eggs injected with saline and different types of NPs.

The effect of in ovo injection of different NPs types on the body weight (BW) of ducks at day-hatch and at 7 days of age was evaluated and compared with saline-injected and non-injected control groups, as shown in Fig. 4. The results clearly demonstrated that the type of NPs played a significant role in influencing the duckling weight. Figure 4 shows significant increases in BW on the day-old hatchlings and the 7th day of the tested ducks hatched from eggs injected with NPs compared to the control, indicating the effective impact of the prepared NPs on the growth performance in general. Ducks hatched from eggs injected with Fe_3_O_4_-NPs, ZnO-NPs, and Cu-NPs exhibited significantly higher body weights at 7 days of age than those in the non-treated control group, with increases of approximately 28.95%, 28.36%, and 20.28%, respectively. Moreover, remarkable and significant increases in BW on the old day were observed for groups treated with the prepared NPs in comparison with the control and saline groups, indicating the positive effectiveness of the injection of NPs on the BW of the ducks on the old day.

The best BW at the old and 7th day was observed in groups treated with Fe_3_O_4_-NPs and ZnO-NPs, followed by those injected with Cu-NPs. While the control and saline-treated ducks showed comparable body weights, indicating that the injection procedure and saline itself had no adverse or growth-promoting effects, all NPs-treated groups demonstrated enhanced growth, suggesting a specific biological effect attributable to the prepared NPs. Among the NPs treatments, Fe_3_O_4_-NPs and ZnO-NPs led to the highest increase in BW. This trend may be explained by the known physiological roles of zinc as an essential trace element involved in enzymatic activity, protein synthesis, immune function, antioxidant defense, and immune development, all of which are essential for early growth in poultry^16^. Zinc is critical for growth regulation and intestinal development, which may enhance nutrient absorption during the early post-hatch period^68^. Similarly, copper plays a crucial role in energy metabolism, erythropoiesis, and connective tissue development, and Cu-NPs have been shown to improve growth performance and feed utilization in broiler chickens^69^. The significant increase observed with Fe_3_O_4_-NPs may be associated with improved iron availability, which supports hemoglobin synthesis and oxygen transport, thereby enhancing metabolic efficiency^10,11^. Importantly, the absence of growth suppression or weight loss in NPs-treated groups indicates that the applied doses were non-toxic and well tolerated during embryonic development and early post-hatch life. The similar body weights between the control and saline groups further confirmed that the observed growth enhancement was specifically related to NPs exposure rather than injection stress.

These findings are consistent with the observations of^70^, who demonstrated that zinc supplementation significantly enhanced the growth performance of broiler chickens subjected to heat stress. Moreover, ZnO-NPs are promising alternatives to conventional zinc sources in poultry nutrition. Their increasing interest is attributed to their distinctive physicochemical properties, including a high specific surface area, enhanced adsorption capacity, and superior catalytic activity, which may improve zinc bioavailability and physiological utilization compared with traditional zinc supplements^71^. This supports the assumption that the observed increases in the NPs-treated groups were attributable to the biological activity of the prepared NPs rather than the mechanical or osmotic effects of in ovo manipulation^33^.

#### Blood parameters

##### Serum lipid profile and liver enzymes activities

The effects of in ovo injection of Fe_3_O_4_-NPs, Cu-NPs, and ZnO-NPs during embryogenesis on the serum lipid profile and liver enzyme activities of 7-day-old Domiaty duck chicks were evaluated (Table 1). The results obtained illustrated that embryonic exposure to the prepared NPs significantly modulated lipid metabolism and hepatic enzyme activities during early post-hatch growth. Injection of NPs significantly decreased serum cholesterol, TG, and LDL levels in the ducks. Additionally, the saline group exhibited the highest concentrations of cholesterol, TG, and LDL, followed by the non-injected control group, whereas the lowest values were recorded in the Fe_3_O_4_-NPs group, followed by Cu-NPs and ZnO-NPs. These results indicate a remarkable effect of reducing blood lipid levels when NPs are administered during embryonic development, which may persist into the early post-hatching period of life. In contrast, serum HDL levels were significantly increased by in ovo injection of NPs compared with both control groups, with the highest HDL level observed in ducks obtained from Fe_3_O_4_-NPs-injected eggs. An increase in HDL is considered a beneficial metabolic adaptation because HDL plays a critical role in reverse cholesterol transport and in maintaining lipid homeostasis. Similar results were reported by^22^, who observed a significant elevation in HDL levels following in ovo injection of Fe_3_O_4_-NPs. The change in lipid metabolism as a result of NPs treatment may be attributed to their role in enhancing the enzymatic activity involved in lipid transport and oxidation. However, iron homeostasis is critical, as excessive iron accumulation may alter lipid metabolism through oxidative modification of cholesterol fractions^72^. Moreover, reduced serum cholesterol concentrations may reflect decreased hepatic cholesterol synthesis or increased catabolism and excretion, leading to enhanced HDL levels^73^.

Regarding liver function biomarkers, ALT, AST, and ALP activities were evaluated to assess the hepatic response in 7-day-old ducklings, as listed in Table 1. Significant differences were detected in ALT and ALP activities among the experimental groups (*P* < 0.05), whereas AST activity was not significantly affected (*P* > 0.05). Ducks from the control and saline groups exhibited significantly lower ALT and ALP activities than those from the NPs-treated groups. In contrast, in ovo injection with NPs, especially Cu-NPs and ZnO-NPs, resulted in moderate but significant improvements in ALT and ALP activities. These changes remained within physiological ranges, indicating enhanced hepatic metabolic activity rather than hepatocellular damage^74^. ALT and ALP are commonly used indicators of liver metabolic function, and their moderate increase may reflect increased protein synthesis, mineral metabolism, and enzymatic activity associated with rapid growth during the early post-hatching period^75^.

In addition, no significant changes were observed in AST activity across all treatments, revealing that in ovo NPs injection did not induce liver tissue damage in ducks at 7 days of age. These findings are consistent with those of previous studies reporting improved hepatic enzyme activity following dietary or in ovo supplementation with trace elements, such as zinc. Organic zinc supplementation enhances serum ALP activity in broiler chickens, supporting the role of zinc in liver function, skeletal development, and enzyme regulation^76,77^.

**Table 1:** Effect of in ovo injection of NPs during embryogenesis on serum lipid profile and liver enzyme activity in Domiaty duck strain.

Treatment	Liver %	Chol. mg/dL	TG mg/dL	HDL mg/dL	LDL mg/dL	ALT U/L	AST U/L	ALP U/100 mL
Control	2.16	182.40^b^	93.80^ab^	47.80^b^	115.84^b^	16.56^b^	47.20	181.20^b^
Saline	2.55	199.20^a^	102.00^a^	45.40^b^	133.40^a^	16.52^b^	48.00	180.20^b^
Fe_3_O_4_-NPs	3.86	157.80^c^	80.80^b^	64.00^a^	77.64^c^	17.40^ab^	50.00	191.80^ab^
Cu-NPs	3.71	156.00^c^	81.00^b^	60.60^a^	79.20^c^	17.94^a^	49.80	197.20^a^
ZnO-NPs	3.26	166.60^c^	82.40^b^	63.80^a^	86.32^c^	17.70^a^	48.60	195.40^a^
SEM	0.53	4.921	5.485	2.84	4.66	0.335	1.381	3.904
P. value	0.187	< 0.0001	0.0429	0.0001	< 0.0001	0.0188	0.5758	0.0129

a-c. Means within a variable with no common subscripts differ significantly (*P* ≤ 0.05). Cholesterol (Chol.); triglycerides (TG); high density lipoprotein (HDL); Low density lipoprotein (LDL); alanine (ALT); aspartate transaminase (AST); and alkaline phosphatase (ALP).

##### Serum immunoglobulin G, transferrin, hemoglobin, and thyroid hormones

The results listed in Table 2 show that in ovo injection of metal-based NPs, including Fe_3_O_4_-NPs, ZnO-NPs, and Cu-NPs, during embryogenesis affected the hematological parameters, thyroid hormone profiles, and humoral immunity in Domiaty duck strain. Regarding hemoglobin and transferrin, the observed significant increases in hemoglobin (Hb) and transferrin (Tf) concentrations, specifically in the Fe_3_O_4_-NPs group, revealed the superior bioavailability of iron oxide when administered in nanoparticulate form. Iron is a fundamental component of hemoglobin synthesis and erythropoiesis, and its delivery at the nanoscale may enhance its uptake and cellular utilization owing to the increased surface area and reactivity^78,79^. Moreover, Fe_3_O_4_-NPs may reduce heme breakdown in the spleen and enhance the lifespan of red blood cells, contributing to higher hemoglobin levels^14^. The enhanced Hb levels observed after ZnO-NPs injection may be due to the higher bioavailability and cellular uptake of nanostructured Zn and its essential role in the activity of enzymes in the heme synthesis pathway. Transferrin binds trace metals, such as zinc; therefore, in ovo injection with ZnO-NPs can improve iron binding and transport dynamics, possibly affecting transferrin circulation, as listed in Table 2^80^. Additionally, the higher Hb and Tf concentrations recorded in the Cu-NPs group compared with the control group may be attributed to the synergistic role of copper in iron metabolism, including stimulation of ceruloplasmin activity, iron mobilization, and hemoglobin synthesis^81^. Similar findings were reported by^69^, who demonstrated that Cu-NPs exhibited higher bioefficacy than inorganic copper salts, leading to improved growth performance and hematological indices in broilers.

Thyroid hormones are critical regulators of embryonic growth, energy metabolism, and thermoregulation in avian species^82^. The significant increases in serum triiodothyronine (T3), thyroxine (T4), and T3/T4 ratio in NPs-treated groups compared to the control and saline groups confirmed the crucial role of the prepared NPs in enhancing thyroid activity and metabolic regulation. T3 levels were significantly increased as a result of in ovo injection with Fe_3_O_4_-NPs, Cu-NPs, and ZnO-NPs by 74.79, 68.38%, and 50.43%, respectively, compared to the control groups. The higher T3 levels observed in the NPs groups may reflect improved peripheral conversion of T4 to the biologically active T3 form, indicating enhanced deiodinase enzyme activity. Similarly, serum T4 concentration was significantly increased in the groups injected with Fe_3_O_4_-NPs, Cu-NPs, and ZnO-NPs compared to the control group by 31.38, 31.21, and 26.17%, respectively. Zinc and copper are known cofactors for thyroid hormone metabolism and antioxidant defense systems, which may explain the pronounced response in the ZnO-NPs and Cu-NPs groups^83^. Additionally, the positive relationship between blood T3 hormone concentrations and oxygen consumption supports the hypothesis that NPs enhance metabolic efficiency and growth potential in the early post-hatch life^84^.

**Table 2:** Effects of in ovo injection of NPs during embryogenesis on serum transferrin, hemoglobin, Immunoglobulins G, and thyroid hormones levels in the Domiaty duck strain.

Treatments	TF mg/10 ml	Hb g/100 ml	IgA mg/dl	IgM mg/dl	IgG mg/dl	T3 ng/ml	T4 ng/ml	T3/T4 ratio
Control	18.64^c^	11.38^c^	45.60	121.20	158.00^b^	2.34^c^	5.96^b^	0.40^b^
Saline	22.41^bc^	11.20^c^	47.40	118.20	158.20^b^	2.40^c^	6.18^b^	0.39^b^
Fe_3_O_4_-NPs	32.76^a^	16.88^a^	47.20	124.60	184.20^a^	4.09^a^	7.83^a^	0.52^a^
Cu-NPs	22.55^bc^	14.62 ^b^	51.40	132.60	192.60^a^	3.94^ab^	7.82^a^	0.51^a^
ZnO-NPs	25.15^b^	15.62^ab^	48.00	131.00	190.00^a^	3.52^b^	7.52^a^	0.47^ab^
SEM	1.959	0.460	2.619	4.675	3.877	0.162	0.245	0.026
P. value	0.0009	< 0.0001	0.6229	0.1781	< 0.0001	< 0.0001	< 0.0001	0.0041

a-c. Means within a variable with no common subscripts differ significantly (*P* ≤ 0.05). Hemoglobin (Hb); Transferrin (TF); Immunoglobulins G (IgG, IgA and IgM); Tri-iodothyronine (T3) and Thyroxine (T4).

While serum IgA and IgM levels were not significantly affected by in ovo NPs treatment, a significant increase in IgG concentration was observed, as shown in Table 2, particularly in the Cu-NPs and ZnO-NPs groups. IgG plays a central role in systemic humoral immunity and passive immunoprotection. Zinc is essential for lymphocyte proliferation, antibody production, and cytokine signaling, and its nanoparticle form may further enhance immune responsiveness owing to improved cellular uptake^16^. The enhanced IgG response observed in Cu-NPs-treated ducklings may be attributed to their role in immune cell metabolism and antioxidant defense^85^. ZnO-NPs and Fe_3_O_4_-NPs also contributed to IgG increase; however, their effects were comparatively lower than those of Cu-NPs.

In general, these results indicate that in ovo injection of the prepared metal NPs is an effective strategy for improving hematological status, endocrine function, and humoral immunity in the tested ducks. Among the tested NPs, Fe_3_O_4_-NPs primarily enhanced the oxygen-carrying capacity and metabolic hormones, whereas Cu-NPs exerted the strongest immunostimulatory effect, particularly on IgG production. These NPs-specific responses underscore the importance of designing nano-nutrient formulations according to targeted physiological outcomes during embryonic development.

##### Serum and hepatic antioxidant activities

The findings listed in Table 3 clearly illustrate that in ovo injection of Fe_3_O_4_-NPs, Cu-NPs, and ZnO-NPs during embryogenesis significantly changed the antioxidant status of the Domiaty duck strain. The observed enhancement in systemic and hepatic antioxidant capacity indicates that early NPs exposure can promote the antioxidant defense system during critical stages of embryonic development. In ovo injection of the prepared Cu-NPs and ZnO-NPs resulted in a significant increase in total antioxidant capacity (TAC) in the serum compared to the control and saline groups. The Fe_3_O_4_-NPs group showed a remarkable increase in TAC, but not more than that of the Cu-NPs and ZnO-NPs groups. Similarly, TAC levels in the liver tissue were significantly increased by all injections compared to those in the control and saline groups. The highest TAC activity in the liver was observed in the group treated with Cu-NPs, followed by those injected with ZnO-NPs and Fe_3_O_4_-NPs, with increases of 41.30, 40.44, and 30.87%, respectively, compared with the control group. This enhancement reveals improved redox homeostasis and the ability of trace NPs to activate endogenous antioxidant defense. The superior performance of Cu-NPs and ZnO-NPs may be attributed to their direct involvement as structural or catalytic cofactors in key antioxidant enzymes^83^.

Serum superoxide dismutase (SOD) and glutathione (GSH) activities were also significantly increased in all NPs-treated groups compared with the control, indicating an upregulation of enzymatic and non-enzymatic antioxidant mechanisms. Zinc is an essential component of CuZnSOD (SOD1), which accounts for approximately 90% of the total SOD activity and plays a pivotal role in scavenging superoxide radicals and protecting tissues from oxidative damage^86^. Furthermore, Zn-induced metallothionein synthesis enhances antioxidant protection by neutralizing hydroxyl radicals and binding redox-active metals, thereby limiting oxidative stress^87,88^. Cu supplementation has been shown to stimulate SOD activity as part of a multi-layered antioxidant defense strategy that includes limiting free radical generation and preserving mitochondrial integrity^89^. In contrast, malondialdehyde (MDA) concentrations were significantly reduced, specifically in ducks obtained from eggs injected with Cu-NPs and ZnO-NPs, followed by Fe_3_O_4_-NPs, which showed a remarkable decrease compared to the control and saline groups. MDA is a well-established indicator of lipid peroxidation and oxidative membrane damage^90^, and its reduction confirms the ability of NPs injection to mitigate oxidative stress. Similar reductions in MDA levels have been reported in poultry supplemented with ZnO-NPs at a concentration of 60 mg/kg BW, reflecting improved membrane stability and antioxidant efficiency^91^. The increased glutathione (GSH) activity observed in this study may represent an adaptive protective mechanism that compensates for fluctuations in enzymatic antioxidants during the early post-hatching growth stages^92^. In general, the enhancement of TAC, SOD, and GSH, together with the suppression of lipid peroxidation, indicates that in ovo NPs injection effectively strengthens the antioxidant defense system of the Domiaty duck strain, improves oxidative stability, and potentially enhances post-hatch health and physiological adaptation.

**Table 3:** Effect of in ovo injection of prepared NPs during embryogenesis on serum and hepatic antioxidant activities in Domiaty duck strain.

Treatments	TAC mmol/L	MDA µmol/mL	SOD U/ml/h	GSH nmol/ml	TAC liver µmol/g
Control	15.20^b^	8.40^a^	61.60^c^	226.60^c^	4.60^b^
Saline	15.40^b^	8.00^a^	59.60^c^	238.20^c^	4.02^b^
Fe_3_O_4_-NPs	16.80^ab^	7.10^ab^	73.00^b^	252.20^b^	6.02^a^
Cu-NPs	18.80^a^	5.80^b^	80.80^ab^	273.40^a^	6.50^a^
ZnO-NPs	19.20^a^	5.40^b^	83.00^a^	276.00^a^	6.46^a^
SEM	0.897	0.587	3.396	7.831	0.407
P. value	0.0111	0.0056	0.0003	0.0006	0.0006

a-c. Means within a variable with no common subscripts differ significantly (*P* ≤ 0.05). Total antioxidant capacity (TAC); superoxide dismutase (SOD); glutathione (GSH), malondialdehyde (MDA).

### Histological observation

**Fig. 5:**
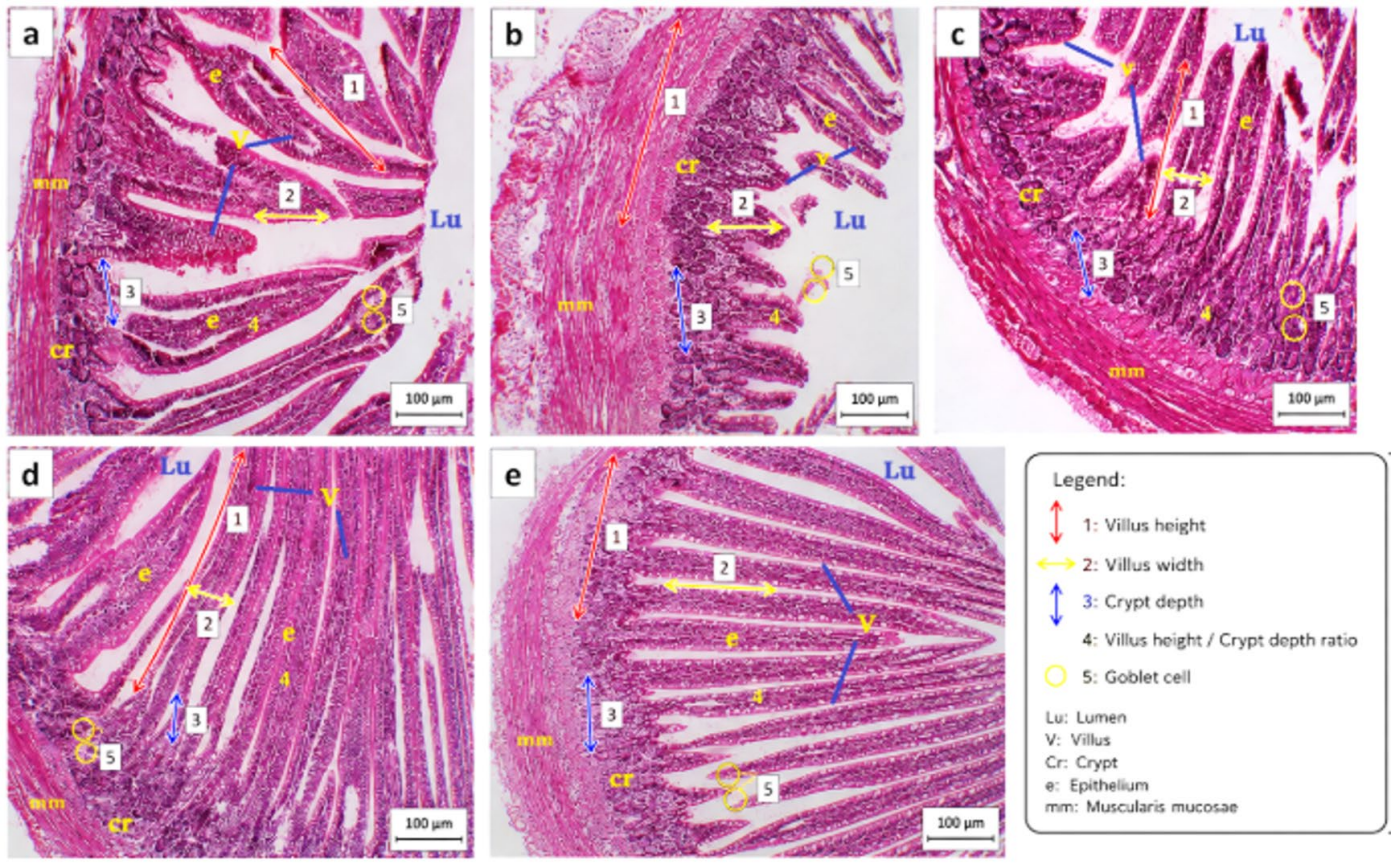
Photomicrographs of jejunal sections showing the effect of in ovo administration of biosynthesized Fe_2_O_3_-NPs, Cu-NPs, and ZnO-NPs on intestinal morphology in Domiaty ducks, including images of the jejunum of: (**a**) control group, (**b**) saline group, (**c**) Fe_3_O_4_-NPs group, (**d**) Cu-NPs group, and (**e**) ZnO-NPs group at a magnification of 10 x, where mm: muscularis mucosa; Lu: Lumen; V: Villi; e: epithelial lining; Cr: Crypts of Lieberkühn.

Microscopic histological examination of the jejunum of the tested Domiaty ducks revealed remarkable alterations in villus architecture and crypt development among the tested groups, illustrating that *in ovo* injection with the prepared NPs influenced intestinal development in the tested ducks. Intestinal morphology is a crucial indicator of gut health and functional maturity, particularly in establishing nutrient absorption and immune efficiency during the early post-hatching period. Similarly, a higher villus height to crypt depth ratio reflects a more mature intestinal energy expenditure for tissue renewal and a greater capacity for nutrient assimilation^93^. Figure 5 shows that ducks in the control and saline groups exhibited relatively short and narrow villi with fewer goblet cells and less developed crypts of Lieberkühn. Similar morphological features are typically related to a reduction in the absorption surface area and limited digestive efficiency in the absence of prepared NPs during embryogenesis.

The intestinal mucosa is the primary site of nutrient digestion and absorption, and its structural development during the early post-hatch period is closely associated with subsequent growth and physiological performance. Improved intestinal architecture may increase the effective absorptive surface area and facilitate nutrient utilization during this critical developmental stage^94^.

In contrast, *in ovo* injection of Fe_3_O_4_-NPs, Cu-NPs, and ZnO-NPs resulted in outstanding improvements in jejunal histoarchitecture, including increased villus height and diameter, greater villus density per microscopic field, and a higher number of well-developed crypts per microscopic field. These alterations enhance epithelial renewal and improve intestinal function. Among the NPs-treated groups, the ZnO-NPs and Cu-NPs groups exhibited the most notable effects, as evidenced by elongated villi and numerous, deeply invaginated crypts embedded within the lamina propria. Increased villus dimensions correspond to a larger absorptive surface area, thereby enhancing nutrient uptake^95^. The remarkable increase in the number and size of crypts in the groups treated with the prepared NPs indicates a higher rate of cellular proliferation and accelerated tissue turnover. The crypts of Lieberkühn are considered proliferative zones responsible for epithelial renewal; therefore, their expansion is an indicator of regenerative activity in the intestinal mucosa^96^. Zinc and copper are important cofactors for several enzymes involved in DNA synthesis, antioxidant defense, and epithelial repair processes. Moreover, their small nanoscale size may enhance their cellular uptake and bioavailability during the embryonic stage^97^.

Additionally, the increase in the number of goblet cells and mucus-secreting activity in the groups injected with NPs showed an improvement in mucosal protection and intestinal barrier integrity. Furthermore, mucus secretion plays an important role in the lubrication of digestive tract movement, protecting epithelial cells from pathogens, and enhancing enzyme activity in the brush edge membrane^98^. The remarkable increase in gut mucosal thickness in ducks injected with the prepared NPs during embryonic development improved the rate of digesta passage and nutrient assimilation, leading to superior feed utilization and outstanding growth. In addition, the villus height-to-crypt depth ratio is a recognized indicator of intestinal efficiency and appears to be enhanced by NPs injection, illustrating a shift toward absorptive rather than proliferative activity, which is favorable for optimal digestion and absorption^10^. In general, *in ovo* injection of the prepared NPs positively altered jejunal histomorphology in the tested Domiaty ducks, confirming the effectiveness of *in ovo* injection of the tested NPs as a strategic tool for improving poultry production.

The positive effects observed following in ovo injection of ZnO-NPs, Fe_3_O_4_-NPs, and Cu-NPs may be attributed to their high bioavailability and enhanced cellular uptake. Zinc is essential for maintaining intestinal epithelial integrity, enterocyte proliferation, and regulation of brush border membrane function, which collectively enhance nutrient absorption efficiency. Moreover, zinc participates in the maintenance of tight junction proteins and supports intestinal barrier function, thereby facilitating effective nutrient transport across the intestinal epithelium^99,100^. Iron plays a critical role in cellular respiration, oxygen transport, and energy metabolism, all of which are required for the rapid development of intestinal tissue during embryogenesis. Therefore, adequate iron availability may promote intestinal maturation and improve the gut absorptive capacity. Copper also contributes to intestinal health through its involvement in numerous metalloenzymes associated with antioxidant defense and metabolism. Enhanced copper bioavailability may reduce oxidative stress-induced tissue damage and support normal intestinal development^101,102^. Furthermore, nano-mineral supplementation has been reported to improve intestinal morphometric characteristics, including villus length, villus width, and absorptive surface area, resulting in improved nutrient utilization and growth performance in poultry. Therefore, the enhanced intestinal morphology observed in the present study likely contributed to improved nutrient absorption and physiological performance of the developing embryos and post-hatch ducklings^93,103^.

The findings of the present study support the hypothesis that in ovo administration of biosynthesized Fe_3_O_4_-NPs, ZnO-NPs, and Cu-NPs may improve the physiological performance of Domiaty ducks through integrated gut immune growth. The enhanced bioavailability of nanosized trace minerals, resulting from their small particle size and increased surface area, may facilitate more efficient uptake and tissue utilization during the critical period of late embryonic development^101^.

The improved intestinal morphology observed in the treated groups may have increased the absorptive capacity of the gastrointestinal tract by enlarging the intestinal surface area available for nutrient digestion and absorption. Enhanced intestinal development during embryogenesis has been associated with improved post-hatch nutrient utilization and growth potential^4,5^. Consequently, improved nutrient availability may have contributed to the superior growth performance observed in the nano-mineral-treated group.

The greater body weight recorded in the Fe_3_O_4_-NPs group may be related to the pivotal role of iron in oxygen transport, mitochondrial respiration, and cellular energy metabolism, thereby directing absorbed nutrients toward tissue accretion and growth^104^. ZnO-NPs may support growth through its involvement in intestinal epithelial development, protein synthesis, and metabolic regulation, while also maintaining intestinal barrier integrity^100^.

In contrast, the superior immune response observed in the Cu-NPs group may reflect the preferential role of copper in antioxidant defense and immune regulation. Copper functions as an essential cofactor for several enzymes involved in oxidative stress protection and immune cell activation, whereas improved intestinal integrity may enhance mineral absorption and support the development of gut-associated immune tissue^101^.

Therefore, although improvements in intestinal morphology may represent a common mechanism enhancing the bioavailability and absorption of nano-trace minerals, the distinct physiological functions of iron, zinc and copper appear to determine the predominant biological response. Fe_3_O_4_-NPs preferentially enhanced growth performance, ZnO-NPs contributed to both growth and intestinal health, and Cu-NPs exerted a greater influence on immune competence. Collectively, these findings suggest that in ovo administration of biosynthesized nano-trace minerals may improve the overall physiological performance of Domiaty ducks through coordinated interactions among intestinal development, nutrient absorption, growth, and immune function.

## Conclusion

In conclusion, the present study demonstrates that in ovo administration of green-synthesized Fe_3_O_4_-NPs, Cu-NPs, and ZnO-NPs at 30 µg/egg effectively influenced early growth, physiological status, antioxidant capacity, immune response, and intestinal development in Domiaty ducks. The NPs were successfully synthesized with stable nanoscale characteristics, ensuring their suitability for biological applications. Under the experimental conditions of the present study, embryonic exposure to these NPs improved hatchling body weight, post-hatch performance, hematological indices, thyroid activity, IgG levels, lipid profile, antioxidant status, and intestinal morphology. In particular, Fe_2_O_3_-NPs and ZnO-NPs were associated with greater growth responses, Whereas Cu-NPs showed more pronounced effects on selected immune and antioxidant indicators. The observed improvements in intestinal morphology may have contributed to enhanced nutrient utilization and post-hatch development, whereas the differential responses among the nano-minerals may reflect their distinct biological functions. Overall, the findings indicate that in ovo administration of green synthesized Fe₂O₃-NPs, Cu-NPs, and ZnO-NPs may represent a potential nutritional approach to support early physiological development in Domiaty ducks. Further molecular, biochemical, and long-term production studies are warranted to clarify the mechanisms involved and confirm the broader applicability of this strategy under different production conditions.

## Data Availability

All data supporting the findings of this study are available within the paper.
